# Differences in calculated body fat percentage estimated from published equations based on bioelectric impedance analysis in healthy young South African adults

**DOI:** 10.1177/22799036231196732

**Published:** 2023-09-14

**Authors:** Muhindo Macky Kyusa, Herculina Salome Kruger, Zelda de Lange-Loots

**Affiliations:** 1 Centre of Excellence for Nutrition, North-West University, Potchefstroom, South Africa; 2 Medical Research Council Unit for Hypertension and Cardiovascular Disease, North-West University, Potchefstroom, South Africa

**Keywords:** Obesity, body fat percentage, bioelectric impedance, prediction equations, young adults, South Africa

## Abstract

**Background:**

Adult overweight and obesity, in addition to the intake of saturated fat and total serum cholesterol must be monitored as biological risk factors for non-communicable diseases (NCDs). Bioelectric impedance analysis (BIA) provides data on body fat for use in epidemiological settings. However, optimized equations should be used to calculate percentage body fat (%BF). The purpose of this study was to assess the differences between %BF calculated using different published BIA equations and %BF measured by BIA in young South African adults.

**Design and methods:**

In this observational study, differences in calculated %BF were assessed, with different BIA equations retrieved from the literature used in 1128 healthy young adults aged 20–30 years. The %BF (measured by BIA) was compared between equations, between Black and White men and women, respectively.

**Results:**

The results showed statistically significant differences in the %BF calculated from published BIA equations when used in young South African adults (χ² = 946, χ² = 2528, χ² = 2088, respectively, p < 0.0001). In Black and White men and women, respectively, %BF levels were significantly higher when calculated by equations, than when measured by BIA (p < 0.0001).

**Conclusion:**

There seem to be large discrepancies in estimating %BF by BIA equations and these values cannot be used interchangeably for young South African adults. A South African age, ethnicity and sex-specific BIA equation needs to be developed to accurately estimate %BF in young South African adults.

## Introduction

Non-communicable diseases (NCDs) are the principal causes of death, contributing to more than 73.4% of all deaths worldwide.^1^ The growing prevalence of NCDs and their consequences contribute to an important socioeconomic burden in both high-income countries (HIC) and low- and middle-income countries (LMIC).^2^ Nojilana et al.^3^ estimated that 26% of people aged 30 years would die prematurely in South Africa and recommended that NCDs’ surveillance be strengthened and enabled to furnish reliable information for health policy planning and monitoring. From a set of 25 indicators of the NCDs Global monitoring framework,^4^ obesity in adults should be monitored as a biological risk factor.

Body mass index (BMI) ranges, commonly used to assess obesity, have limitations regarding body fat determination. BMI ranges have led to the misclassification of persons regarding cardiometabolic risk due to adiposity, as demonstrated by the meta-analysis by Deurenberg et al.^5^ Obesity expressed as body fat percentage (%BF) is detected at a lower BMI range than the WHO cut-off points in some populations (ref). The association between BMI and %BF is also not similar among different ethnic groups. BMI considers fat and lean body mass without making any distinction; thus, it intrinsically measures the excess of weight to height instead of the excess of fat.^6^ In contrast, direct %BF measurement involves complex equipment and time demands and is thus not practical for epidemiological studies.^7^ Current bioelectric impedance analysis (BIA) technology has acceptable precision and accuracy compared to other predictive techniques; it is relatively inexpensive, quick to perform, portable, non-invasive and meets the safety requirements for an assessment tool.^8^

Different BIA prediction equations have been used to determine %BF in adults, but they have been developed and validated to predict fat free mass (FFM), lean body mass (LBM), and total body water (TBW) and they customarily only aim to be applied in the studied populations and specific age ranges.^9^ Ethnic or population differences in fat and muscle mass and their implications for BIA, fat mass (FM) and FFM determination have been demonstrated, highlighting the necessity for ethnic-specific prediction equations and cut-off points in the diagnosis of overweight and obesity.^10^ There may be significant differences between these European, Asian, American and African ethnic groups in terms of %BF cut-off point and fat distribution. The purpose of this study was to assess the differences between %BF calculated using different published BIA equations and %BF measured by BIA in young South African adults.

## Design and methods

### Study participants

The study used data from the African-PREDICT study baseline of 1 128 healthy young adults, 574 women (292 White and 282 Black), and 554 men (264 White and 290 Black). The African-PREDICT study baseline was conducted at the Hypertension Research and Training Clinic on the North-West University Potchefstroom campus from 2013 to 2017.^11^

Ethical approval was obtained from the Health Research Ethics Committee of the Faculty of Health Sciences of the North-West University and all participants gave informed consent (NWU-00212-21-S1).

### Variables

The sex, body weight (kg), standing height (m), BIA derived resistance and reactance parameters from the African-PREDICT study participants were reduced to FFM using 10 published equations: Lohman A, Lohman B,^12^ Gray cited by Houtkooper et al.,^13^ Modeling the Epidemiologic Transition Study (METS), METS 2b, METS 3b,^14^ Jakicic et al.,^15^ Lukaski, Deurenberg, and Stolarzyk recommended by Houtkooper et al.^13^ The resulting FFM were converted to %BF using the equation %BF = (Body weight−FFM)/body weight × 100.^13^

### Statistical methods

The differences in calculated %BF, when the different BIA equations retrieved from the literature were used for African-PREDICT participants, were assessed using Friedman’s test with Bonferroni adjustment. The differences between %BF measured by BIA and calculated %BF using BIA equations retrieved from the literature in Black and White men and women, respectively, were performed using the Wilcoxon signed-rank test to determine the differences between %BF obtained for the same participant using the two methods (Bodystat output and calculation from the equation) for each of the different equations. Bland–Altman analysis was performed to determine the agreement between %BF calculated using different equations and %BF measured by BIA. As the %BF estimated by a criterion method remains unknown, the differences between paired data (%BF from predictive equations and %BF from Bodystat^®^) were plotted against averages and the closeness of differences to the mean %BF across the %BF mean ranges were explored to determine the strength of agreement between methods.^16^ Data processing and statistical analysis of data were performed using Statistical Package for the Social Sciences (*SPSS)* (IBM^®^ SPSS Statistics version 27). The level of significance was set at *p* < 0.05 and the confidence at 95% confidence interval (CI).

## Results

The characteristics of the participants are shown in Table 1. Body height, BMI (kg/m²), waist circumference (cm), hip circumference (cm), %BF, lean mass (kg), fat mass (kg), impedance_50 khz (Ω), resistance (Ω), and reactance (Ω) were significantly different between Black and White women. All characteristics were significantly different between Black and White men, except %BF.

**Table 1.** Characteristics of participants (median [IQR]).

Variables		Women (*n* = 574)			Men (*n* = 554)		
		Black (*n* = 282)	White (*n* = 292)	*p*-Value	Black (*n* = 290)	White (*n* = 264)	*p*-Value
Sociodemographic data	Age (year)	24.0 [22.0, 27.0]	24.0 [22.0,27.0]	0.488	24.0 [22.0,27.0]	25.0 [22.0,27.0]	0.170
Anthropometric measurements	Body height (cm)	159.5 [155, 163]	166 [162, 170]	<0.0001	169 [165,174]	179 [175,183]	<0.0001
	Body weight (kg)	65.2 [56.3, 72.5]	64.8 [57.0,72.1]	0.886	61.8 [55.2,72.2]	83.7 [74.8,92.1]	<0.0001
	BMI (kg/m²)	25.5 [21.9, 28.7]	23.0 [20.8,25.6]	<0.0001	21.48 [19.4, 24.2]	26.0 [23.2,28.3]	<0.0001
	Waist circumference (cm)	76.2 [69.0, 84.4]	73.1 [68.4,79.2]	0.005	75.0 [70.0,81.5]	87.2 [81.0,93.4]	<0.0001
	Hip circumference (cm)	105 [97.3, 112]	100 [95.2,106]	<0.0001	92.5 [86.5,99.7]	103 [97.5,109]	<0.0001
	Neck circumference (cm)	31.7 [30.2, 33.0]	31.7 [30.6,33.0]	0.283	35.1 [34.0,36.7]	38.8 [37.0,40.5]	<0.0001
BIA measurements	Bodyfat percentage (%)	33.2 [28.3,38.0]	27.3 [23.5,32.4]	<0.0001	16.3 [13.3,19.9]	16.7 [12.8,22.0]	0.249
	Lean mass (kg)	43.1 [39.1,46.7]	46.4 [42.8,50.1]	<0.0001	52.3 [47.4,59.8]	69.0 [64.3,73.7]	<0.0001
	Fat mass (kg)	21.6 [21.6, 27.0]	17.2 [13.5,22.8]	<0.0001	9.8 [7.6,12.9]	13.95 [13.9,19.8]	<0.0001
	Impedance_50 khz (Ω)	642 [587,711]	631 [578,687]	0.030	567 [506,617]	479 [446, 525]	<0.0001
	Resistance (Ω)	638 [583.0,706]	628 [574,683]	0.039	562 [502,611]	475 [442,521]	<0.0001
	Reactance (Ω)	68.2 [62.4,74.9]	66.4 [61.9,72.0]	0.037	68.8 [63.0,75.0]	61.35 [57.6, 65.7]	<0.0001

### Differences in calculated %BF estimated by the different published BIA equations

The %BF BIA equations retrieved from the literature were classified into three categories. The first category has been developed to calculate %BF for both women and men without distinction. These equations are the ones developed by Lukaski, Deurenberg, and Stolarzyk,^13^ as well as Jakicic et al.^15^ These equations were applied to the total group of participants. The second category was developed to calculate %BF only in women. These BIA equations were developed by Luke et al.,^14^ Lohman,^12^ Sun et al.,^17^ and Gray et al., cited by Houtkooper et al.^13^ and the equations have been applied to data of women. The third category is made up of equations developed to calculate %BF in men only. These equations were developed by Luke et al.,^14^ Lohman,^12^ Sun et al.,^17^ and Gray et al., cited by Houtkooper et al.^13^ All calculated %BF data are presented in Table 2.

**Table 2.** Calculated %BF when the different BIA equations retrieved from the literature are used (median [IQR]).

Men & women combined			Women			Men		
Equations	*N*	Median, IQR	Equations	*N*	Median, IQR	Equations	*N*	Median, IQR
Jakicic et al.^15^	1128	31.3 [25.1, 37.1]	Lohman1992 A^12^	574	34.9 [29.6, 40.3]	Lohman1992 A^12^	554	23.1 [16.7, 29.5]
Lukaski^13^	1128	30.7 [22.4, 37.8]	Lohman1992 B^12^	574	30.5 [25.9, 34.6]	Lohman1992 B^12^	554	18.3 [14.1, 23.4]
Deurenberg^13^	1128	29.6 [22.1, 35.9]	Gray^13^	574	32.2 [27.0, 37.8]	Gray^13^	554	19.7 [14.9, 24.6]
Stolarzyk^13^	1128	33.7 [26.8, 40.0]	Luke2013mets^14^	574	36.7 [32.1, 41.0]	Luke 2013mets^14^	554	21.8 [16.4, 27.7]
			Luke2013mets2b^14^	574	38.4 [33.7, 42.7]	Luke 2013mets2b^14^	554	23.7 [18.4, 29.6]
			Luke2013mets2b^14^	574	35.8 [30.8, 41.1]	Luke 2013mets3b^14^	554	24.2 [18.6, 30.4]

IQR: interquartile range.

The results showed statistically significant differences in the %BF calculated from BIA equations retrieved from the literature (χ² = 945.9, *p* < 0.0001). The %BF medians are presented in Table 2. The lowest median %BF of 29.6% was obtained using the Deurenberg equation,^13^ while the highest median %BF of 33.7% was obtained using the Stolarzyk equation.^13^ After application of the Bonferroni adjustment, the %BF differed significantly among all the equations.

**Figure 1.**
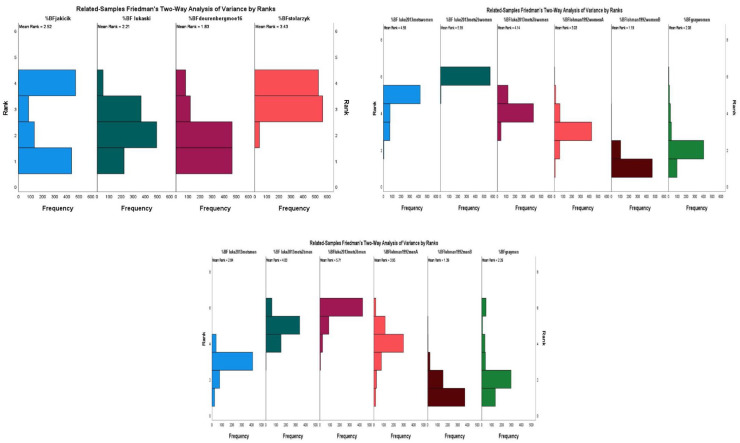
Analysis of variance by ranks of %BF calculated using each of the equations developed to calculate %BF for both women and men without distinction, only in women and only in men.

There were also statistically significant differences in the %BF calculated from BIA equations developed to calculate %BF for women only (χ² = 2528, *p* < 0.0001), as well as for men only (χ² = 2087.7, *p* < 0.0001). The lowest median %BF for women of 30.5% was obtained using the Lohman equation B for women,^12^ while the highest median %BF of 38.4% was obtained using the Luke et al. Mets2b equation.^14^ The lowest median %BF for men of 18.3% was obtained using the Lohman equation B for men,^12^ while the highest median %BF of 24.2% was obtained using the Luke2013mets3b equation.^14^ After the application of the Bonferroni adjustment, the %BF differed significantly among all equations. The %BF medians are presented in Table 2 and the analysis of variance by ranks in Figure 1.

### Differences between %BF measured by BIA and calculated %BF using published BIA equations in Black and White men and women, respectively

**Figure 2.**
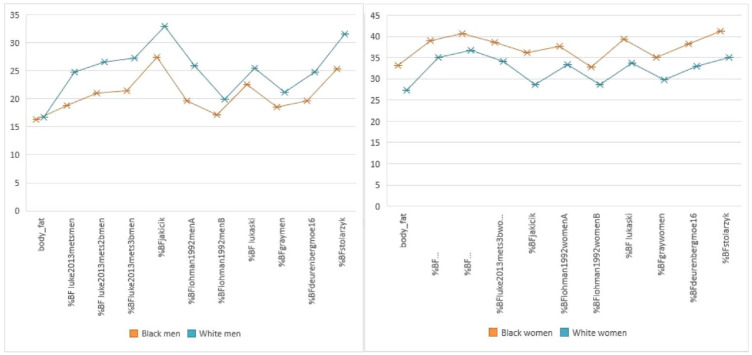
Differences between %BF from equations and BIA in black and white men and women. Y-axis: %BF (%), X-axis: %BF by BIA (Bodystat, Douglas, UK) and the different BIA predictive equations.

In both groups of Black and White men and women, the %BF levels when calculated using any of the equations were significantly higher than when measured by the BIA. The difference between the %BF of Black and White men and women measured by BIA and calculated using different BIA equations are presented in Figure 2.

### Agreement between %BF calculated using different equations with %BF measured by BIA

In general, there is poor agreement between %BF from each equation and the %BF measured by BIA in this study. The %BF calculated from equations were either below or above the %BF measured by BIA. For all four BIA equations that have been developed to calculate %BF for both women and men without any distinction, the calculated %BF was higher than the %BF measured by BIA (Supplemental Figure S1). They all showed a significant systematic difference with BIA equations overestimating %BF compared to the BIA measurements. The scatter plots (Supplemental Figure S1) show differences between –20% and +20% for the different individuals in the study, when two different methods are compared. Differences are not consistent across the range of %BF measured and calculated in this study. Within the range of negative differences, the mean difference ranged between –5% and –10% of %BF. For these BIA equations, there is a lack of agreement between %BF from each equation and the %BF measured by BIA.

For the six BIA equations developed to calculate %BF only in women, the %BF calculated using three equations was higher than the %BF measured by BIA (Supplemental Figure S1). For these equations a significant systematic difference with BIA equations overestimating %BF compared to BIA was observed. However, the %BF calculated using the Lohman B BIA equation yielded on average 0.2% (95% CI 0.0, 0.4) units less than the %BF measured by BIA. A significant systematic difference with BIA equations underestimating %BF compared to BIA was observed. All these BIA equations showed good agreement between %BF from each equation and the %BF measured by BIA. Plots reveal consistent differences across the range of %BF of the individuals. In the range of low %BF (1%–30%) equations seem to overestimate %BF and therefore differences are mostly between –10% and +2%BF. In the range of higher %BF (30%–50%), these methods underestimate %BF and therefore differences are mostly between 0% and 10%.

The %BF calculated using the six BIA equations that were developed to calculate %BF only in men was higher than the %BF measured by BIA (Supplemental Figure S1). They all showed a significant systematic difference from BIA measurements, with equations overestimating %BF. All these BIA equations showed poor agreement between %BF from each equation and the %BF measured by BIA. The poor agreement was consistent across the %BF ranges for all equations, based on consistent differences across the range of mean BF% and several observations falling outside the 95% confidence intervals of the means.

## Discussion

The results have demonstrated significant differences in calculated %BF when the 10 BIA equations retrieved from the literature were used for the African-PREDICT participants. Differences between the %BF measured by BIA and calculated %BF using BIA equations were statistically significant in Black and White men and women, respectively. All equations provided higher %BF than the BIA measurements. Some equations showed a poor agreement, and only a few showed a good agreement with the BIA measurements. Some equations systematically overestimated the %BF compared to BIA measurements, except the Lohman B equation for women.^12^ Differences were not consistent across ranges of %BF and can therefore not be used interchangeably in South African young adults.

The BIA predictive equations were selected based on the fact that they met one of the following criteria: validated for the prediction of %BF, developed in young adult populations, used densitometry as criterion methods and sample included populations of African origin. The Jakicic equation^15^ was included owing to its specificity of being developed in overweight populations. The recommended BIA equations for predicting FFM by Houtkooper et al.^13^ were also included.

Several studies have compared the %BF derived from BIA with %BF measured by more accurate and reliable methods such as dual-energy X-ray absorptiometry (DXA) or air displacement plethysmography (ADP) in young adults.^18–21^ This study focuses on comparing different BIA based predictive equations for the estimation of %BF in young adults and how they behave when applied to young South African adults. When the same equation was applied to White and Black men and women, respectively, the %BF differences remained significant. The set of selected equations for this study encompassed those developed in American and European young white populations and Black Africans. Sinaga et al.^22^ reported that the Caucasian-developed %BF predictive equations significantly underestimate %BF in Ethiopian populations by 6.78%, and that %BF underestimation was more pronounced in men than in women. Body fat percentage predictive equations developed in American and European white populations showed significant differences in predicting %BF in White South African participants. In a similar way %BF predictive equations developed in Black populations showed significant differences in Black South Africans. Differences in ethnicity-related body shape and build, water distribution, musculoskeletal mass, fat patterns, body density, and proportional limb length resulting from differences in body parts like the head, trunk, arms and legs between ethnic groups have been demonstrated.^23–26^ The trunk, arms and legs contribute highly to the generation of the resistance at the basis of the BIA methods. Jakicic et al.^15^ earlier reported the effect of ethnicity on the accuracy of %BF BIA prediction accuracy in obese adult women.

This study also demonstrated the difference in %BF when the same equations are applied in white and black South Africans. Luke et al.^14^ reported that when validating FFM BIA equations in young adults from five populations of African origin, the mean difference was less than 1% between the FFM derived from the developed equations and FFM measured by isotope dilution. The equations developed using data from all five populations combined predicted better agreement than the population-specific equations. In contrast, differences between estimated %BF of young Black and White female South African adults are large enough to justify different equations to calculate %BF in black and white female groups.

The Jakicic equation^15^ was developed in overweight populations and may have limited value in underweight and normal weight groups. It seems to be an outlier in men, possible because we applied it in the total group and not in overweight/obese men only.

The differences between young Black and White male South African adults are also large enough and different equations are required to calculate %BF in Black and White men. Sun et al.^17^ developed a race-combined BIA predictive equation that was more accurate and precise than the race-specific equations. Dioum et al.^26^ highlighted that the magnitude of the predictive error of an equation related to the ethnicity in which the equation was developed and validated and recommended further fundamental research on the determinant of such discrepancies. Even though the conclusion referred to the estimation of total body water, this is also valuable for %BF assessment, as BIA primarily measures body water, while FFM and %BF are calculated from the body water. Dehghan and Merchant^27^ indicated that a %BF BIA prediction equation developed and validated in a population different than the one within which the %BF is being measured, should be calibrated accounting for ethnicity. The study conducted by Deurenberg et al.^23^ concluded that general BIA predictive equations should be avoided across different populations unless validation is performed in those populations. Notwithstanding the above, Bosy-Westphal et al.^28^ suggested that population specificity was of minor importance as regards the discrepancies between different criterion methods.

The study sample in which current equations have been validated may be of concern. The sample representativeness of %BF equation validation studies have an impact on the predictive power of the developed equations. Nationally representative %BF BIA predictive equations have been developed in the USA using NHANES data.^29^ The sample size of studies for the development of the selected equations as applied in the current study ranged from 50 to 200 participants. The small sample size might have accounted for differences in %BF calculated among published equations and between the percentage estimated by BIA.

Four of the selected %BF BIA prediction equations in this study were developed and validated in a general sample without any distinction of sex. Predictive errors are reported to be different when the same equation is applied to men and women. When comparing %BF using a multifrequency BIA, Sun et al.^17^ found that the mean %BF measured by BIA was significantly lower than the mean %BF from DXA, with BIA underestimating and overestimating %BF differently in men. The coefficient for correlation was 0.78 in men and 0.85 in women.

The median BMI differed between Black and White men and women, respectively. FFM measurements in our study are converted to %BF and can present a bias across BMI ranges.^30^ Comparison of FFM derived from BIA and DXA demonstrated a higher concordance across the BMI range, but BIA overestimated FFM from 3.28 to 8.38 kg in the range of BMI >18 and<40 kg/m^2^, while differences were less than 1 kg for BMI between 16 and 18.5 kg/m^2^, and difference varied with BMI for ranges ≥40 kg/m^2^.^31^ Sun et al.^17^ found that the patterns of DXA and BIA were significantly different across %BF ranges. In women, %BF measured by DXA and BIA fitted well within the normal %BF category, while BIA overestimated %BF by 3.6% for lean participants and underestimated %BF by 2.6% for obese participants. In men, DXA and BIA presented small differences in normal %BF, while BIA overestimated %BF by 3% for lean participants and underestimated %BF by 4.3% for obese participants. The fact that the current study’s participants span all the ranges of obesity up to a BMI of 35 kg/m^2^ in this study, and that the mean BMI in the validation samples of selected studies seems to be different from the current study, could contribute to differences of %BF among different selected BIA predictive equations.

Most of the predictive equations used to derive %BF in this study were developed to estimate FFM and not %BF. Brewer et al.^19^ found that when comparing body composition estimates from the MF-BIA and the 4C model, the prediction error of a BIA equation can differ when using the same equation to derive FFM or %BF. The greatest %BF error was found in women. It is now known that the validity of FFM BIA predictive equations should not be reported when the resulting FFM is converted to %BF.^29^ This could have contributed to the lack of agreement between equations when these equations are applied to estimate %BF in South African young adults.

Parameters that constitute independent variables in %BF BIA predictive equations could account to some extent for the differences among equations. Völgyi et al.^31^ reported a difference of %BF generated by two BIA devices (InBody 720 and Tanita BC 418 MA) when investigating the effect of obesity, physical activity and age on body composition assessment by BIA and DXA, resulting in the non-integration of age in the InBody 720 built-in equation. The parameters in selected BIA equations were most often height, resistance, sex and age. The Bodystat built-in equation accounts for more parameters; namely sex, age, weight, level of activity, waist and hip circumference but not for height. The differences in the included independent variables may have contributed to the differences between the predicted %BF from the equations retrieved in the literature and %BF estimated by the Bodystat and the resulting poor agreement.

When investigating the agreement among BIA devices based on single frequency (SF) and multi-frequency (MF) methods, Carrion et al.^32^ found differences in %BF measured by devices despite the strong agreement and concordance. The study concluded the same device should be used consistently for reliable %BF measurement. However, Randhawa et al.^33^ reported the absence of differences in %BF using three different devices in a controlled BIA measurement procedure, even when some BIA standard operating procedures were violated in relation to hydration, exercise, water and food intake. Even though BIA devices seem to be relatively precise and reliable for estimating FFM,^34^ attention should be given to their calibration in the population under study. Kutáč and Kopecký suggested that BIA devices should not be used interchangeably, even when they are from the same manufacturer.^35^

Limitations of this study include that, selected equations in this study were developed and validated using different criterion methods; namely deuterium dilution, densitometry and a multi-compartment model (deuterium dilution method, DXA and hydrostatic weighing). This may have contributed to the %BF differences between equations. Nevertheless, Bosy-Westphal et al.^28^ reported non-significant bias between different reference methods when attempting to improve the BIA technology to reduce limitations related to the population specificity of BIA equations. This study assessed the agreement between the selected BIA equations and BIA (Bodystat) for the estimation of %BF. Due to all the factors discussed above, there can be considerable discrepancies between the two methods, with an unacceptable lack of agreement. For some equations, differences are beyond the level of bias of mean difference ± 2 standard deviations (SD) and for others, the trends of the fair agreement are not consistent across the ranges of %BF. Researchers suggested that a maximum prediction error of 1.5–1.8 kg FFM in women and 2.0–2.5 kg in men and an actual error of 0.0–1.8 kg is acceptable. Prediction errors of less than 2.3 kg for women and 3.0 kg for men are very good.^13^ This is clinically important and therefore Bodystat measurements and the different BIA equations tested here cannot be used interchangeably in young South African adults. We cannot recommend one specific equation because %BF was not compared to a criterion method in this study, due to COVID restrictions limiting access to study participants.

In summary, large differences were found between the %BF of young South African adults calculated by BIA published equations and %BF measured by the Bodystat. Different values of %BF were calculated from equations in Black and White groups and in comparison, to BIA. Therefore, the equations cannot be used interchangeably to calculate %BF in young South African adults. This highlights the necessity to develop an ethnicity and sex-specific BIA equation to accurately calculate %BF in young South African adults. %BF calculated by each of the targeted BIA equations should be tested against %BF estimated by a criterion method in young South African adults. The effect size of differences between %BF calculated from equations and measured by a criterion method will determine which equation from those in the literature are recommended for young South African adults.

## Significance for public health

The generated knowledge has highlighted how %BF prediction equations from the literature could be suitable for young, White and Black South African adult women and men, contributing to the accurate measurement of %BF. The discrepancy between the %BF from prediction equations and from BIA highlights the need to develop, and validate a new specific %BF prediction equation that will enable timely and reliable information for health policy planning and monitoring regarding NCDs and their consequences. The risks related to obesity and NCDs can accurately be measured in clinical and epidemiological settings at an affordable cost. This is particularly relevant because of the high prevalence of obesity and NCDs in South Africa and worldwide.

● Equation derived %BF overestimated adiposity compared to BIA.
● %BF equations cannot be used interchangeably for young South African adults.
● Age, race and sex-specific equations need to be developed to estimate %BF.

## Supplemental Material

sj-docx-1-phj-10.1177_22799036231196732 – Supplemental material for Differences in calculated body fat percentage estimated from published equations based on bioelectric impedance analysis in healthy young South African adults Click here for additional data file. Supplemental material, sj-docx-1-phj-10.1177_22799036231196732 for Differences in calculated body fat percentage estimated from published equations based on bioelectric impedance analysis in healthy young South African adults by Muhindo Macky Kyusa, Herculina Salome Kruger and Zelda de Lange-Loots in Journal of Public Health Research
