# Supplementary material for: Differences in calculated body fat percentage estimated from published equations based on bioelectric impedance analysis in healthy young South African adults
Source: J Public Health Res. 2023 Sep 14;12(3):22799036231196732. doi: 10.1177/22799036231196732 (PMC10503279; doi:10.1177/22799036231196732)
Supplement: sj-docx-1-phj-10.1177_22799036231196732 – Supplemental material for Differences in calculated body fat percentage estimated from published equations based on bioelectric impedance analysis in healthy young South African adults [file sj-docx-1-phj-10.1177_22799036231196732.docx]

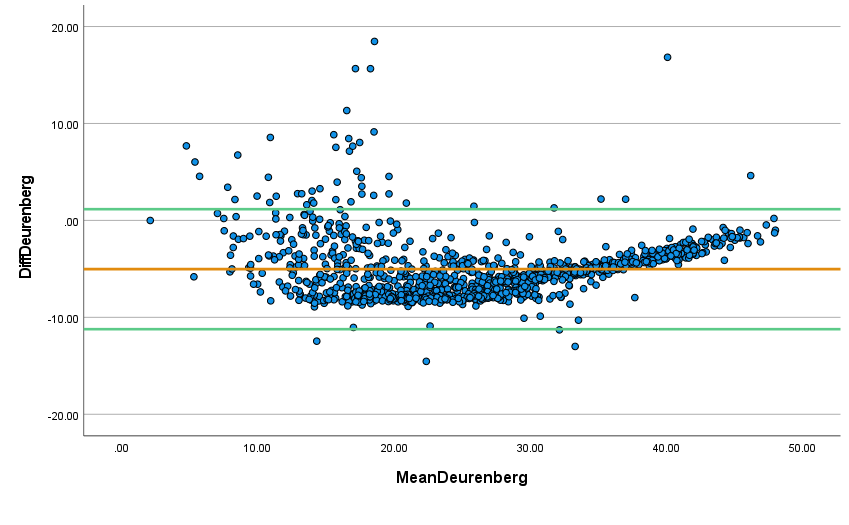

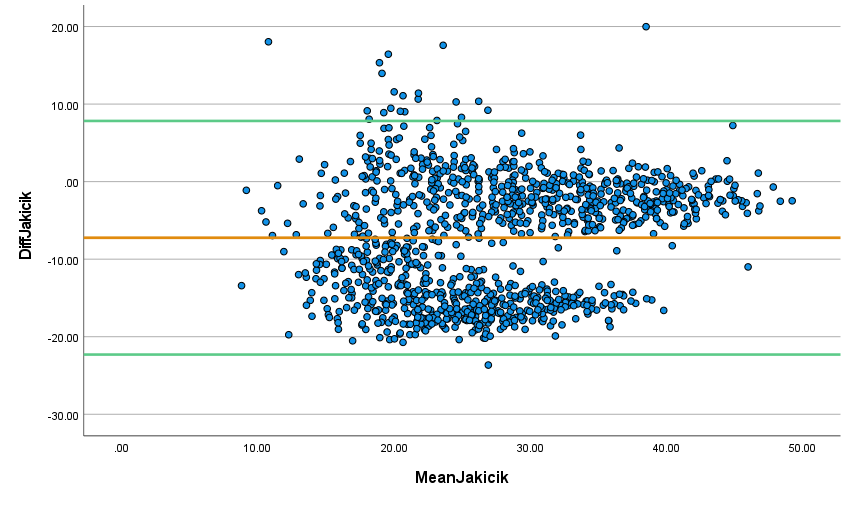

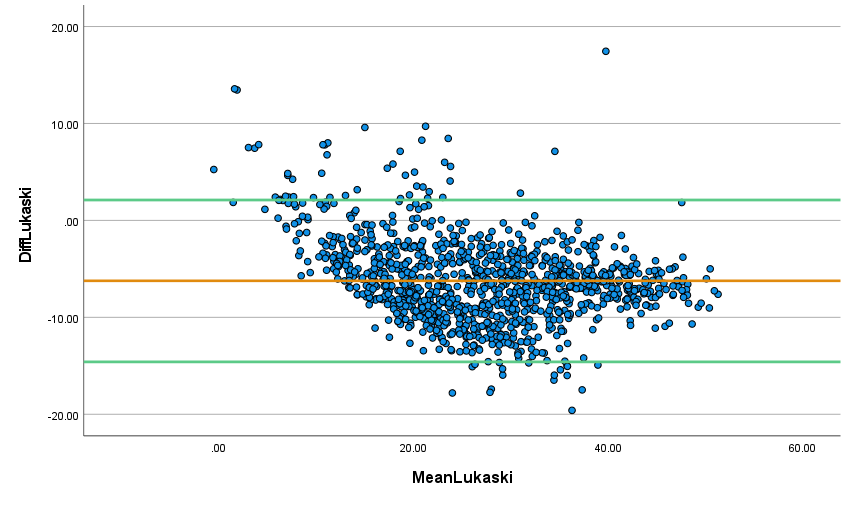

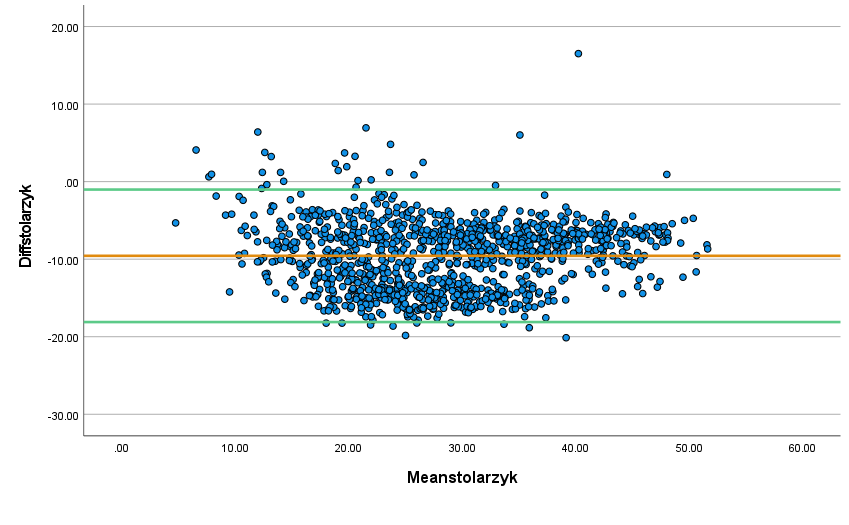


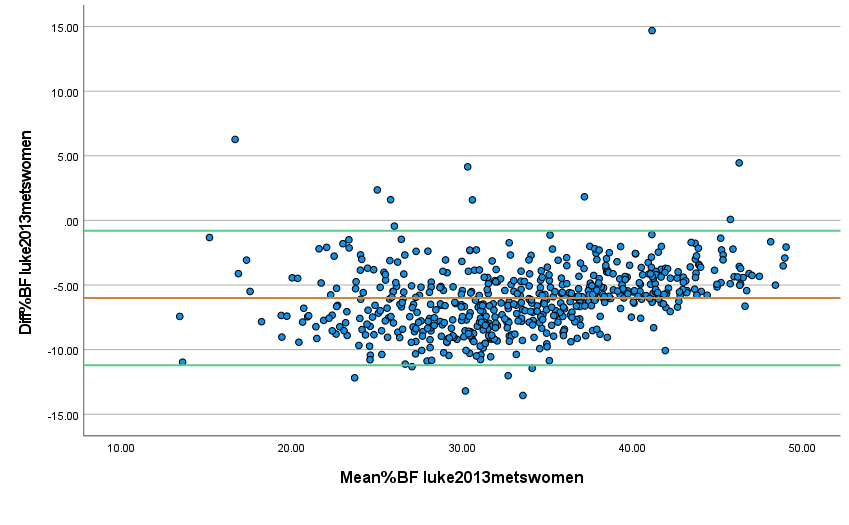

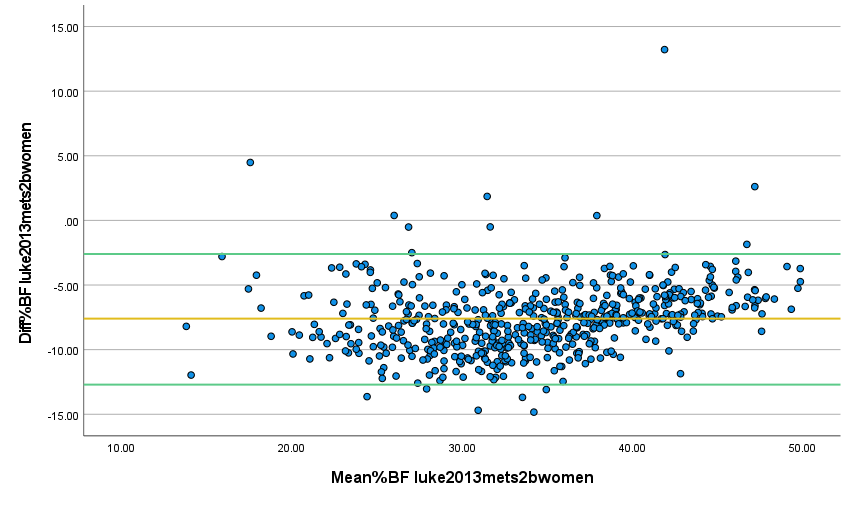

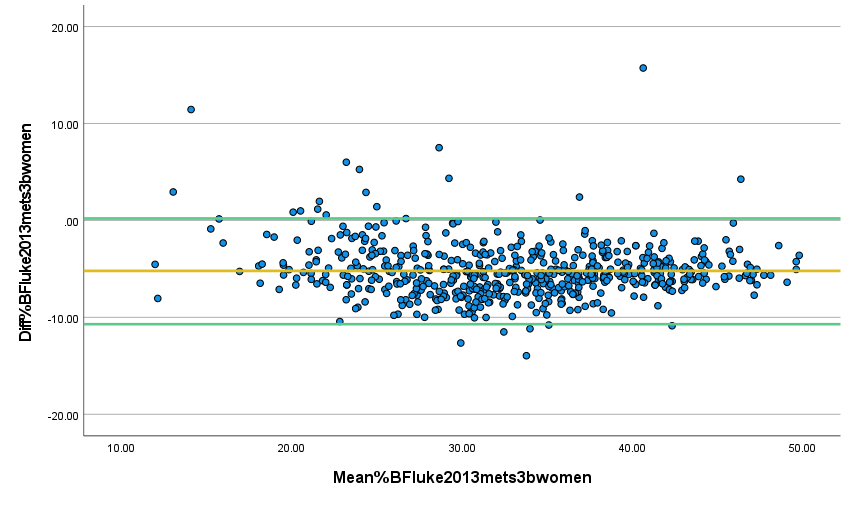


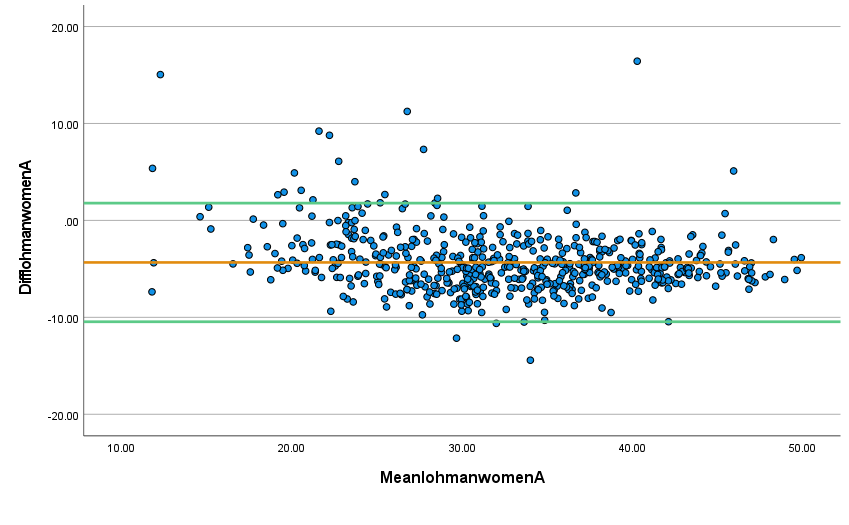

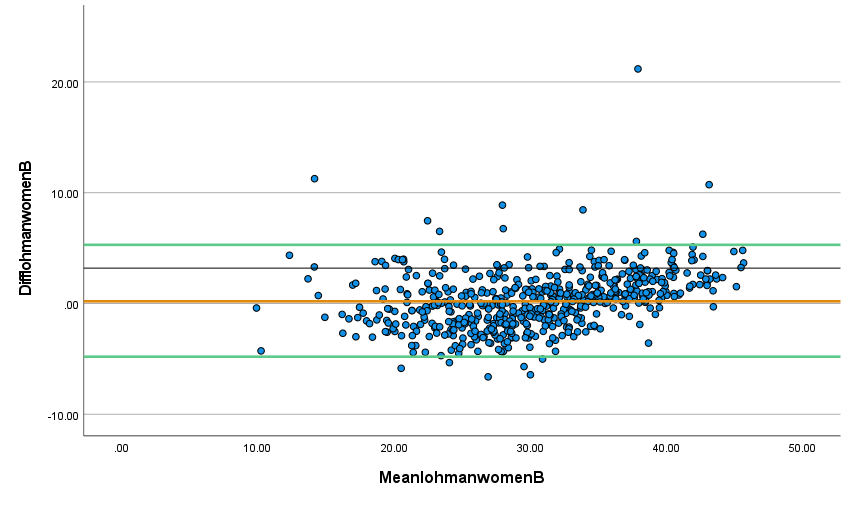

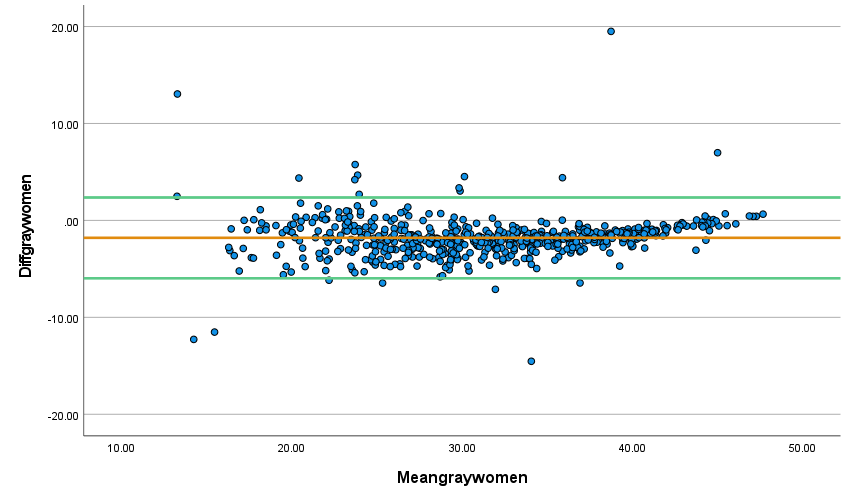


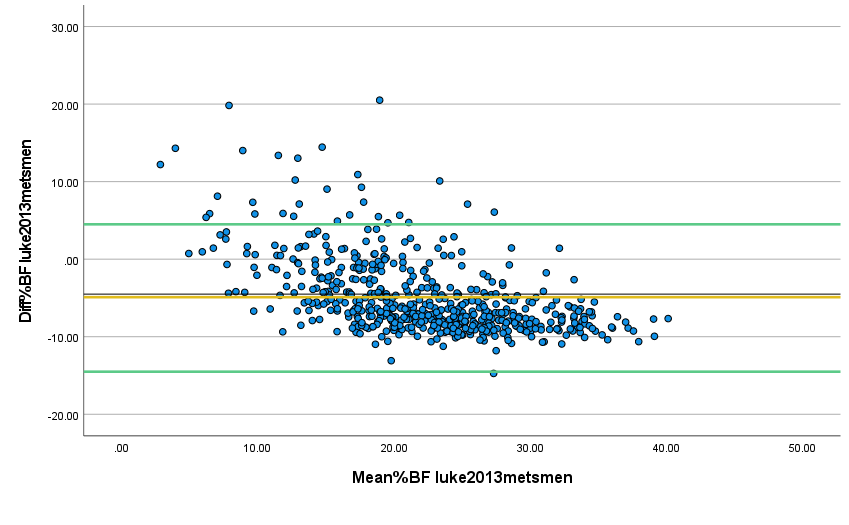

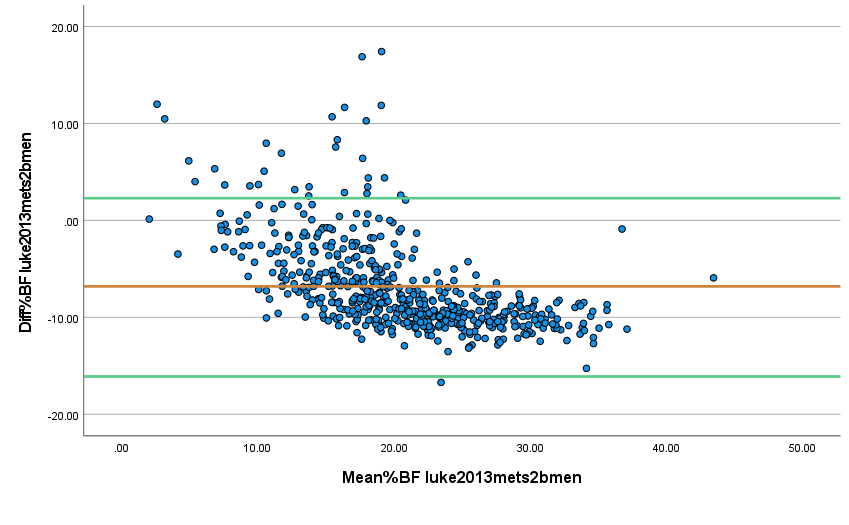

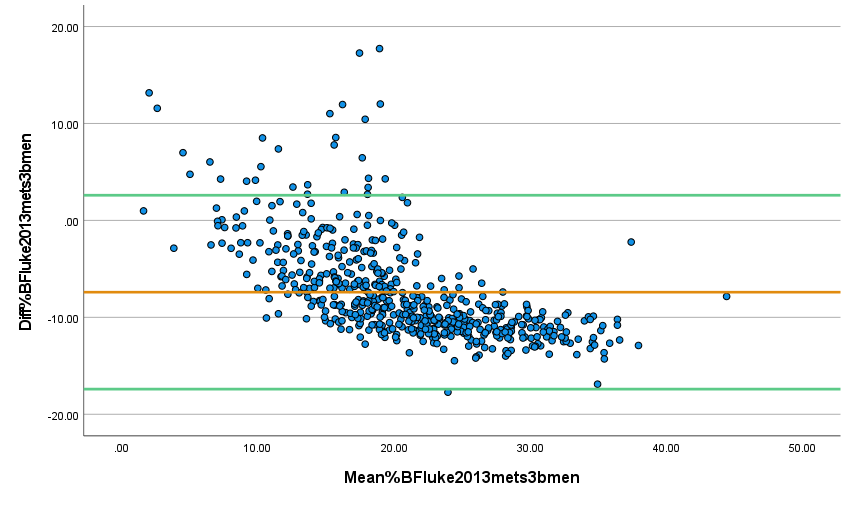

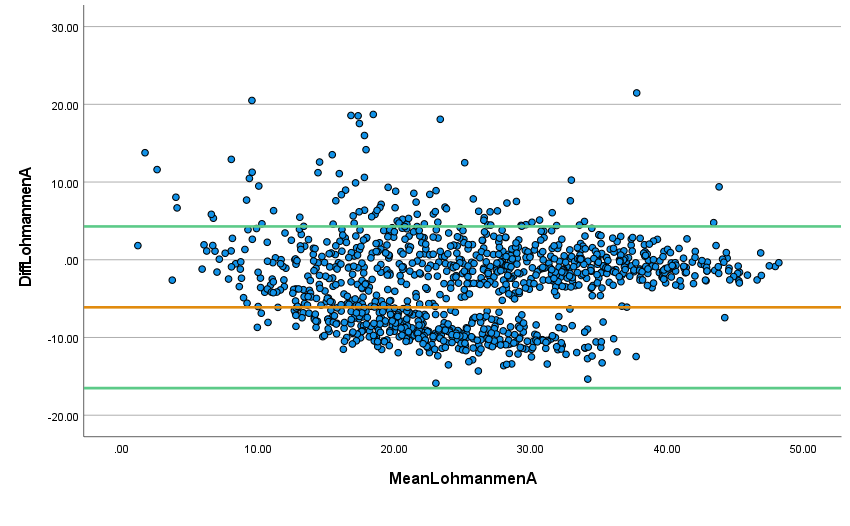

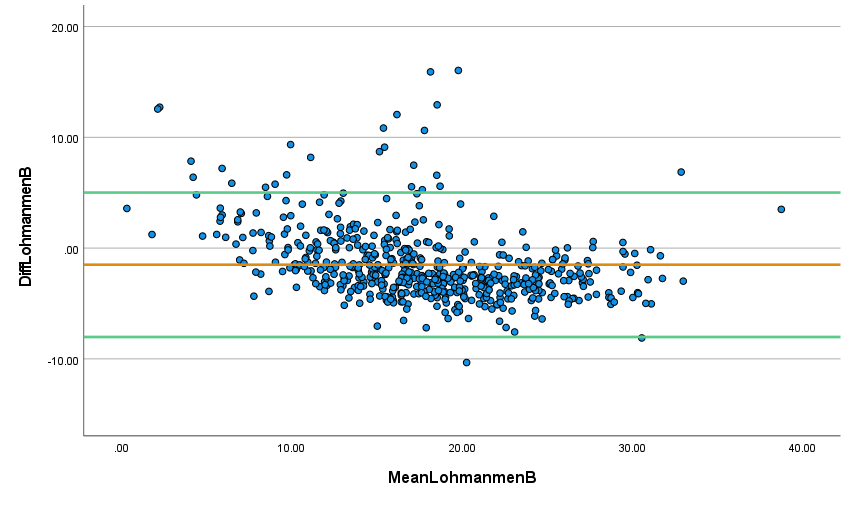

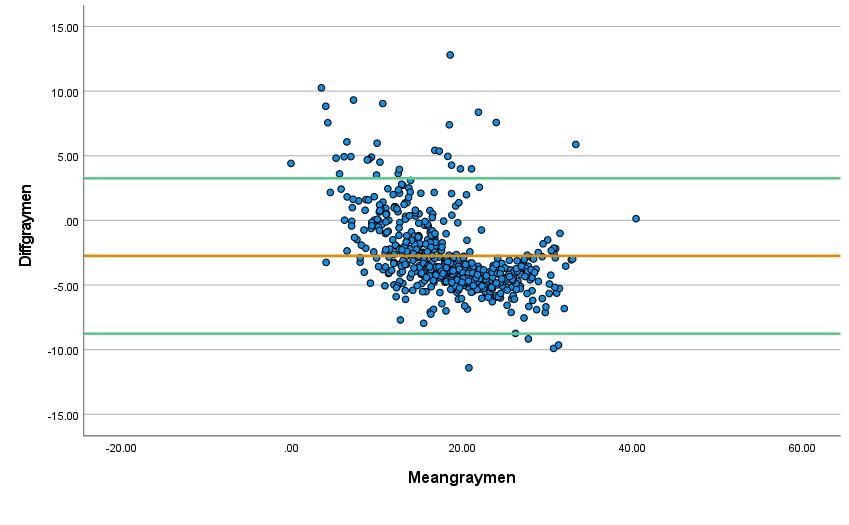


Figure S1: Agreement between the BIA equations developed to calculate %BF for both women and men combined, for women for men, and %BF (%) measured by BIA (Bodystat). Orange lines indicate the mean difference and green lines indicate 95% confidence intervals of the difference
